# Crystal Structure of a Human Single Domain Antibody Dimer Formed through V_H_-V_H_ Non-Covalent Interactions

**DOI:** 10.1371/journal.pone.0030149

**Published:** 2012-01-12

**Authors:** Toya Nath Baral, Shi-Yu Chao, Shenghua Li, Jamshid Tanha, Mehdi Arbabi-Ghahroudi, Jianbing Zhang, Shuying Wang

**Affiliations:** 1 Institute for Biological Sciences, National Research Council Canada, Ottawa, Ontario, Canada; 2 Department of Microbiology and Immunology, College of Medicine, National Cheng Kung University, Tainan, Taiwan; 3 Center of Infectious Disease and Signaling Research, National Cheng Kung University, Tainan, Taiwan; Monash University, Australia

## Abstract

Single-domain antibodies (sdAbs) derived from human V_H_ are considered to be less soluble and prone to aggregate which makes it difficult to determine the crystal structures. In this study, we isolated and characterized two anti-human epidermal growth factor receptor-2 (HER2) sdAbs, Gr3 and Gr6, from a synthetic human V_H_ phage display library. Size exclusion chromatography and surface plasmon resonance analyses demonstrated that Gr3 is a monomer, but that Gr6 is a strict dimer. To understand this different molecular behavior, we solved the crystal structure of Gr6 to 1.6 Å resolution. The crystal structure revealed that the homodimer assembly of Gr6 closely mimics the V_H_-V_L_ heterodimer of immunoglobulin variable domains and the dimerization interface is dominated by hydrophobic interactions.

## Introduction

A typical antibody consists of two heavy-chains (H) and two light-chains (L) [1]. The N-terminal domains of both H- and L-chains are variable and are called variable regions [2], abbreviated as V_H_ and V_L_. Functionally, the antibodies are consisted of an antigen binding domain, Fab and an effector domain, Fc. The Fab is composed of light chain and heavy chain (reviewed by [3]). The antigen binding sites of conventional antibodies contain six complementary determining regions (CDRs), three of them from V_H_ and three from V_L_. The natural minimal antigen binding domain of such antibodies is composed of both V_H_ and V_L_. In camelidae, a significant proportion of functional antibodies are heavy-chain antibodies which do not contain light chain [4]. The antigen binding domain of these heavy-chain antibodies is composed of only V_H_ and is designated as V_H_H (reviewed by [5]). This discovery made it possible to isolate soluble and functional V_H_H-single domain antibody (sdAbs) [6]. These sdAbs have many desirable properties from an antibody engineering point of view. They are relatively small in size with molecular weight of ∼13 kDa and can be engineered to have very high affinities [7]. They can also be amplified and cloned easily because they are encoded by a single gene. In addition, these sdAbs have favorable refolding properties and biophysical stability [8]. Furthermore, they recognize epitopes that are inaccessible to conventional antibodies [9], [10], [11]. Finally, sdAb which is injected intravenously into mice localizes preferentially at the tumor site [12].

Similar types of sdAbs that are derived from human V_H_ [13], [14] are promising in particular for their potential use in immunotherapy because of their human origin. However, the solubility of these human sdAbs is one of the main problems. Several approaches have been reported to obtain soluble V_H_ sdAbs [13], [15], nevertheless structural information of such sdAbs is limited. The absence of light chain leads to exposure of the hydrophobic V_H_-V_L_ interacting interface which can cause aggregation [16]. Hence, the structural information of such human V_H_ sdAbs is very limited. In this regard, we used a synthetic human V_H_ library [17] to isolate a panel of soluble sdAbs against human epidermal growth factor receptor-2 (HER2). The isolated sdAbs have affinities in the nanomolar range. We chose two sdAbs, Gr3 and Gr6, for further evaluation. The difference of the amino acid sequences between these sdAbs is restricted to their CDR1 and CDR3. Expression levels of both sdAbs as soluble protein were comparable. Size exclusion chromatography (SEC) analysis demonstrated that Gr3 exists as a monomer, whereas Gr6 is a dimer. To our knowledge, Gr6 is the first human-derived sdAb that is a strict homodimer. Therefore, we determined the crystal structure of Gr6 which showed that the structure mimics the V_H_-V_L_ pairing.

## Results

### Selection of HER2-specific sdAbs

A human V_H_ phage display library [15], [17] was used to select HER2-binding sdAbs as described [18] with the exception that the first two rounds of panning were performed on MDA-MB-231-Erb2 cells and the third and fourth round on the HER2/Fc protein. Ninety six randomly picked clones were tested on phage ELISA to identify clones displaying HER2-specific V_H_, of which 25 scored positive. DNA sequencing of the 25 clones revealed 7 different V_H_s, namely Gr1, Gr2, Gr3, Gr4, Gr5, Gr6 and Gr7. Gr1 was represented by 12 clones, Gr2 by 4 clones, Gr3 by 3 clones, Gr4 and Gr5 by 2 clones, and Gr6 and Gr7 by 1 clone.

### Characterization of the sdAbs Gr3 and Gr6

**Figure 1:**
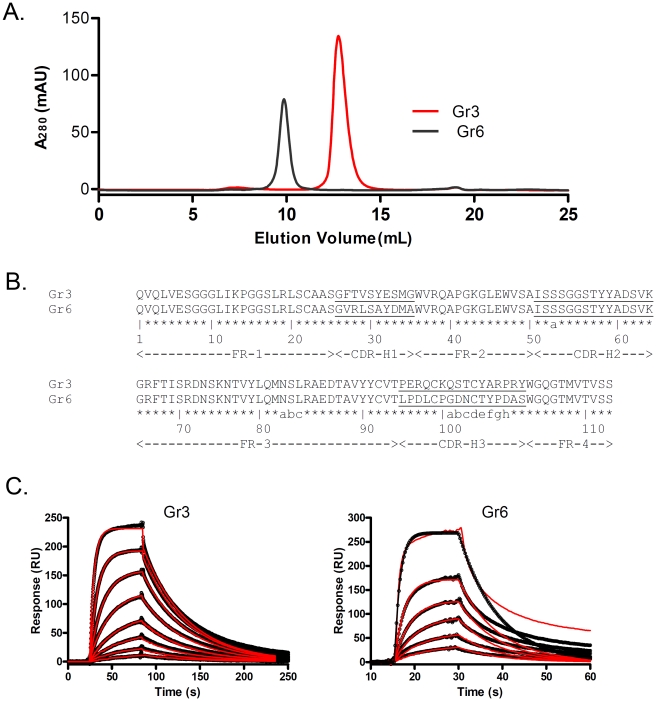
Characterization of Gr3 and Gr6 in solution. (**A**) Size exclusion chromatography of IMAC-purified Gr3 and Gr6 using a Superdex™ 75 column. (**B**) Amino acid sequences of Gr3 and Gr6 numbered according Kabat numbering [19]. (**C**) Binding of Gr3 and Gr6 to HER2/Fc analyzed by SPR.

The seven different V_H_s were sub-cloned in the expression vector pSJF2H [17]. After sequence verification, the sdAbs were expressed as 6xHIS-tagged soluble protein in the periplasm and purified by IMAC using a Ni-NTA column. This one-step purification resulted in more than 95% pure protein when assessed with SDS-PAGE (data not shown). The expression of Gr1, Gr2, Gr4, Gr5 and Gr7 was low and therefore were not used further. Like most of the soluble human sdAbs, Gr3 exists as a pure monomer seen as a single peak eluted at a volume corresponding to the apparent molecular mass of ∼ 15 kDa in a SEC analysis using a Superdex™ 75 column (**Fig. 1A**). However, Gr6 which eluted at a volume that corresponds to the apparent molecular mass of ∼ 30 kDa. This result demonstrated that Gr6 exists as a dimer in solution. Because of this difference, we chose Gr3 and Gr6, which differ only in CDR1 and CDR3 in their amino acid sequences (**Fig. 1B**.) for further characterization.

The affinity of these sdAbs to HER2 was determined with surface plasmon resonance (SPR). Injection of sdAbs at concentration of 5 to 600 nM onto HER2-coupled surface revealed specific binding of the sdAb to the antigen. For Gr3, the actual association and dissociation curves fitted excellently to the 1∶1 Langmuir binding model, giving an association rate constant (*k_a_*) of 2.9×10^5^ M^−1^s^−1^, a dissociation rate constant (*k_d_*) of 2.0×10^−2 ^s^−1^ and a dissociation equilibrium constant (*K_D_*) of 6.8×10^−8^ M (**Fig. 1C**). For Gr6, the *k_a_*, *k_d_* and *K_D_* were 4.5×10^5^ M^−1^s^−1^, 1.0×10^−1 ^s^−1^ and 2.2×10^−7^ M, respectively (**Fig. 1C**).

**Figure 2:**
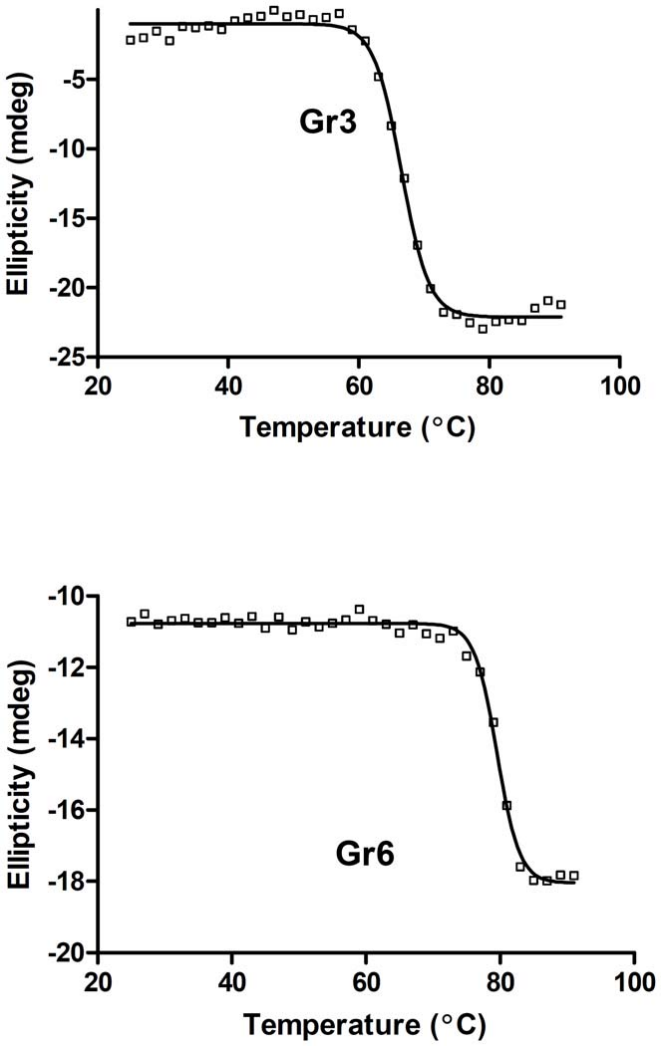
Melting temperature (T_m_) measurements of Gr3 and Gr6 by CD spectroscopy. Melting temperature (T_m_) measurements of Gr3 and Gr6 by CD spectroscopy. The CD values measured at 217 nm are plotted against temperature ranging from 25°C to 91°C. The solid line shows the fitted values using GraphPad Prism software (GraphPad Software Inc., La Jolla, CA).

Circular dichroism (CD) profiles of Gr3 and Gr6 were determined to estimate the thermostability of the sdAbs. Thermo-induced denaturation of the protein was measured in the temperature range of 25 to 91 °C with 2 °C intervals. Plotting the CD value at 217 nm against temperature suggested a two phase denaturation (**Fig. 2**) with a calculated melting temperature (T_m_) of 66 °C for Gr3 and 79 °C for Gr6, respectively.

### Crystal structure of Gr6

**Figure 3:**
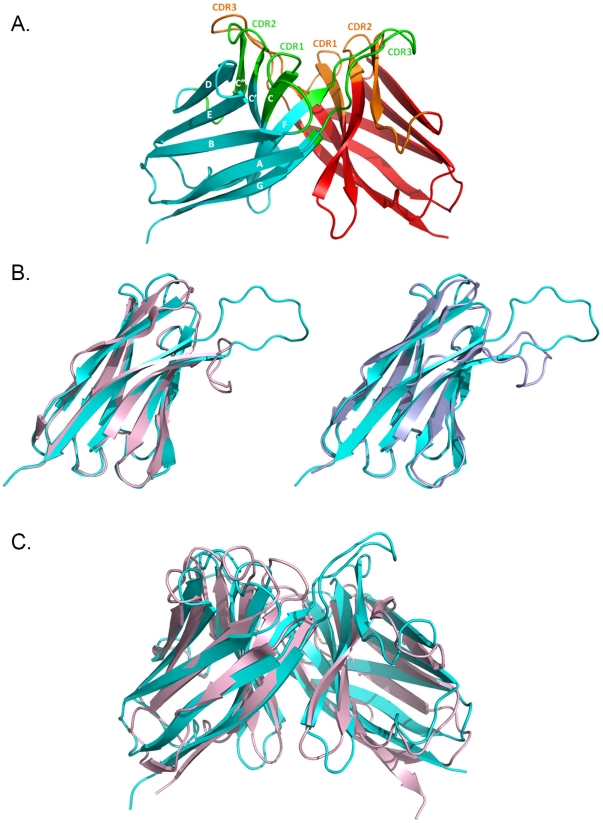
Structure of Gr6 and comparison of Gr6 with cV_H_-E2 and F_v_-POT. (**A**) Ribbon diagram of the Gr6 structure shown in cyan (chain A) and red (chain B). CDRs are colored in green in chain A and orange in chain B. The β-strands are labeled by following the Kabat nomenclature. (**B**) Ribbon diagram of the superimposed F**_v_**-POT Heavy chain onto the monomer of Gr6 (left) and the superimposed cV**_H_**-E2 monomer onto Gr6 monomer (right): cyan, Gr6; light pink, F**_v_**-POT heavy chain; light blue, cV**_H_**-E2. (**C**) Ribbon diagram of the superimposed F**_v_**-POT and Gr6 dimer: light pink, F**_v_**-POT; cyan, Gr6.

Gr6 is a dimeric protein of 140-amino-acid residues and the crystal structure has been solved to 1.6 Å resolution by molecular replacement method (**Fig. 3A**). The majority of the electron density is well-defined which allows for unambiguously model building. The first three residues at the N-terminus in monomer A and the first four residues in monomer B are disordered in the map. In addition, the C-terminus of the last 12 residues in monomer A and 13 residues in monomer B, as well as the 6xHIS tag, could not be traced in the map. Kabat nomenclature [19] is used to number the positions of the amino acids and for the description of the structure. The crystallographic analysis showed that two molecules are present in the asymmetric unit in which one molecule is related to the other by a non-crystallographic 2-fold axis to form a dimer. The refined model includes residues Val2-Gly114 in monomer A and residues Gln3-Ser113 in monomer B as well as 266 water molecules. The residues for β -strand assignments are: 3-12 (strand A), 17-25 (strand B), 32-40 (strand C), 44-52 (strand C’), 56-60 (strand C”), 66-73 (strand D), 76-82b (strand E), 87-96 (strand F), and 102-112 (strand G). Validation of the structure by program SFCHECK [20] showed that the refined model is of good stereochemical quality (Table 1) and that no phi-psi angles are in the disallowed regions of the Ramachandran map.

**Table 1:** Crystallographic data and refinement statistics.

Data collection	
Space group	P2_1_2_1_2_1_
Cell dimensions	
*a*, *b*, *c* (Å)	39.81, 76.99, 81.36
α, β, γ (°)	90.0, 90.0, 90.0
Wavelength	1.000
Resolution (Å)	20.0 – 1.6 (1.66 – 1.60)
*R*_merge_ a	0.052 (0.525)
I/σI	42.5 (3.0)
Completeness (%)	99.4 (99.0)
Redundancy	6.4 (6.3)

Refinement	
No. reflections	32282
*R*_work_ b/*R*_free_ c	20.91/22.89
No. atoms	
Protein	1832
Water	266
*B*-factors	
Protein	25.43
R.m.s. deviations	
Bond lengths (Å)	0.0047
Bond angles (°)	1.37

The values in parenthesis are for the highest resolution bin.

a *R*_merge_  =  *R*_work_  =  Σ_h_Σ_i_|I_hi_ - h>|/Σ_h_Σ_i_I_hi_, were I_hi_ is the *i*th observation of the reflection h, while h> is the mean intensity of reflection h.

b *R*_factor_  =  Σ||F_o_| - |F_c_||/|F_o_|. *R*_free_ was calculated with a small fraction (6.7%) of randomly selected reflections.

c The *R*_free_ was calculated from 6.7% of all data that were not used in the refinement.

The monomer structure of Gr6 has the same fold as the canonical immunoglobulin variable domain, consisting of two β -sheets with five and four strands. Gr6 shares 76% sequence identity with the camelized human antibody fragment cV_H_-E2, an inhibitor of the NS3 serine protease of hepatitis C virus [21] (PDB code 1OL0), and 62% identity with the V_H_ domain of the human Fv-POT [22] (PDB code 1IGM). Superimposition (**Fig. 3B**) of the monomer structure of Gr6 to the monomer structure of cV_H_-E2 and to the V_H_ domain of human Fv-POT yields the root mean square deviation of 0.60 Å and 0.58 Å (117 and 102 C_α_ atoms, respectively). The conformations of the hypervariable loops CDR1 (residues 26-35) and CDR2 (residues 51-64) of Gr6 are similar to those of cV_H_-E2 and Fv-POT. The CDR3 loop (residues 95-102) of Gr6 exhibits a very different structure from the CDR3 loops of cV_H_-E2 and Fv-POT. A unique feature of the Gr6 structure is that CDR3 protrudes beyond the boundary of dimer interface and points to the other side of the other monomer.

Analysis of the dimer interface and the accessible surface area (ASA) was computed by PISA in CCP4 program package [23] and through the Protein Interactions Calculator website (http://crick.mbu.iisc.ernet.in/~PIC) [24]. The total buried ASA at the interface of approximately 2200 Å^2^ is contributed from both monomers of ∼ 1100 Å^2^. The interactions between monomers are predominantly by hydrophobic contacts involving residues Val37, Leu45, Trp47, Ala50, Tyr58, Tyr91, Val93, Leu95, Pro96, Leu98, Ala101 and Trp103. Polar interaction in the interface includes 9 direct hydrogen bonds. No salt bridges and water molecules are found throughout the interface. The arrangement of the Gr6 homodimer resembles the structure of Fv-POT. Superimposition structure of Gr6 with structure of Fv-POT [22] (PDB code 1IGM) reveals that two V_H_ domains in Gr6 structure maintain the same relative orientation between V_H_ and V_L_ in the structure of Fv-POT **(** **Fig. 3C** **)**.

## Discussion

Single-domain antibodies are very attractive tumor-targeting tools for their natural properties of small size, solubility and high permeability into tissues. An important feature of camelid sdAbs is their high thermodynamic stability [8]. This feature not only contributes to the high expression yield, but may also be required for their *in vivo* tumor targeting ability [25]. In this study, we isolated and characterized two anti-HER2 sdAbs, Gr3 and Gr6, from a semi-synthetic human V_H_ library. The T_m_s calculated from CD experiments for both Gr3 and Gr6 are in the same range as of that of the camelid sdAb. Therefore, we presume that these human sdAb are as stable as camelid sdAb. The single domain antibodies are also known for their monomeric behavior. In solution, Gr3 exists as a monomer; however, Gr6 has an unusual nature of being a strict dimer. To our knowledge, Gr6 is the first sdAb derived from human V_H_ with this characteristics and the solved crystal structure of Gr6 presented here is the first structure of human sdAb with a dimeric conformation.

**Figure 4:**
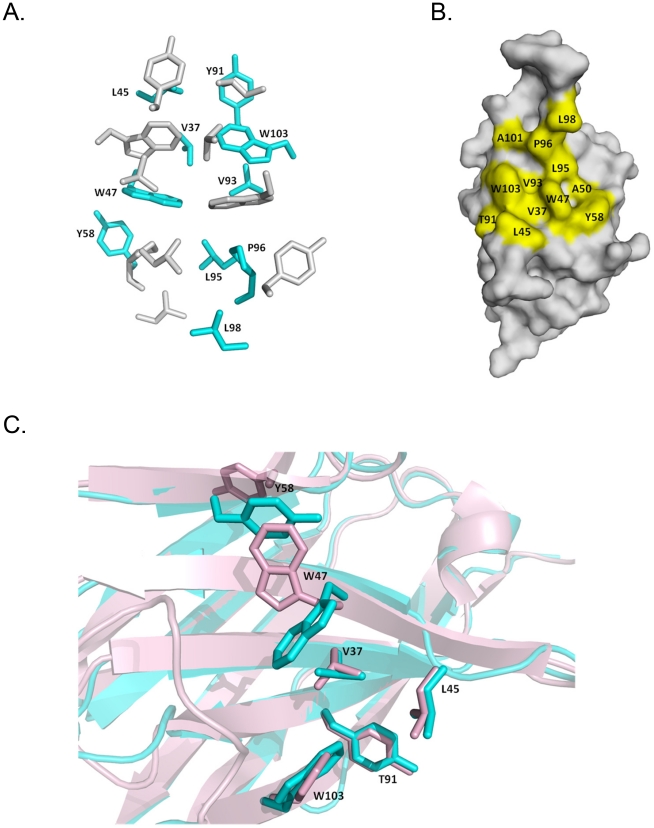
Hydrophobic interface of Gr6 dimer. (**A**) View of side chain arrangement at the V_H_-V_H_ hydrophobic interface of Gr6 homodimer. One Gr6 monomer is shown in cyan and the other one is shown in gray. (**B**) Surface representation of V_H_. Residues involved in dimer formation are highlighted in yellow. (**C**) Superposition of residues at V_H_-V_H_ interface of Gr6 with that at V_H_-V_L_ interface of Fv-POT to show the structural resemblances of the residues that are located at framework-2 involved in the dimer formation.

The crystallographic data presented here shows that Gr6 exists as a homodimer, consistent with the SEC analysis result. The framework structure of Gr6 is identical to other immunoglobulin variable domains, composed of nine β-strands forming two β-sheets. The dimer interface of Gr6 structure is contributed by hydrophobic interaction formed by residues located on β-strands F and G, part of the CDR3 loop and β -strands C and C” (**Fig. 4A and 4B**). Among those residues, Val37, Leu45, Trp47, Ala50, Trp103 are the hall mark residues situated at the V_H_-V_L_ heterodimer interface of the immunologlobulin variable domain [19], [26], [27]. Superposition of these hallmark residues of Gr6 with that of Fv-POT shows structural resemblances **(** **Fig. 4C** **)** that suggest the Gr6 dimer association mimics V_H_-V_L_ interaction.

Interestingly, among all residues contributing to Gr6 dimer formation, eight of them, Val37, leu45, Trp47, Ala50, Tyr58, Tyr91, Val93 and Trp103, are identical between Gr3 and Gr6. The first three amino acids, Val37, Leu45 and Trp47 are substituted to Phe37, Arg45 and Gly47 in camelid V_H_H and are considered to be hallmark substitutions that make these sdAbs more soluble [28], [29]. However, Gr3 which contains all of these three hydrophobic residues is monomeric and soluble.

The other four residues that contribute to the Gr6 dimerization, Leu95, Pro96, Leu98 and Ala101 positioned on CDR3 loop are unique to Gr6. This suggests that these residues play essential roles in Gr6 dimer formation and replacement of these residues in Gr3 (Leu95-Pro, Pro96-Glu, Leu98-Gln, Ala101-Arg) disrupts the inter-domain interaction. It is interesting to note here a change in the character of CDR3 in Gr3 and Gr6. It is hydrophobic in Gr6 but polar/charged in Gr3, thus promoting the change from a dimer to a monomer. This result provides at least an exception of the suggestion that solubility and stability of V_H_ is independent of the CDR3 sequence [16].

The observed relatively large buried ASA ∼ 2200 Å^2^ suggests that the Gr6 structure is a compact dimer. The camelized human antibody fragment cV_H_-E2, a competitive and reversible inhibitor of the NS3 protease of the hepatitis C virus, is a concentration-dependent dimer which occurs in solution at protein concentration > 7 mg mL^−1^ [21]. The dimerization of cV_H_-E2 should be the consequence of the selected CDR3 loop because the molecule was affinity-selected from a library in which CDR3 is randomized, implying that CDR3 should be involved in the dimerization [21]. However, in the crystal structure of cV_H_-E2 dimer, the CDR3 loops are at the opposite sides of the molecule and do not participate in the inter-domain interaction [21]. Therefore, the crystallographic structure of cV_H_-E2 is probably a result of crystal packing and may not represent the observed dimer in solution. In this work, the Gr6 crystal structure shows that the V_H_-V_H_ interface mimics the classical association of V_H_-V_L_ dimer, strongly suggesting that Gr6 is a strict dimer and that the structure does represent the dimerization in solution.

The Gr6 structure present here not only provides information on how the dimer is associated, but also confirms that the dimerization of the sdAb cannot avoid the traditional V_L_-binding interface. More studies are required to understand whether Gr3/Gr6 can block the dimerization of HER2, whether Gr3/Gr6 can specifically target high HER2 expression cells, and how Gr3/Gr6 interacts with HER2. Although trastuzumab (Herceptin, Genentech Inc., San Francisco, CA) is an approved therapy for treating HER-2 dependent cancers, the development of trastuzumab resistance occurred within one year after the patients received the initial response to trastuzumab [30]. One of the problems with trastuzumab is that it does not efficiently block HER2 from dimerizing with other HER family members. Therefore, Gr3 and Gr6 could be alternative HER2-targeted antibodies that possess the advantage of accessibility to hidden antigens that are not easily reached by whole antibodies. The characterization and structural information obtained in this work therefore will be beneficial to the future design of such humanized sdAbs in the HER2-dependent cancer therapy.

## Materials and Methods

### Selection of binders

The synthetic phagemid-based human V_H_ library used in this study has been previously described [15], [17]. V_H_ repertoire was expressed on phage after infection with M13K07 helper phage as described [18]. Specific V_H_s against HER2 were enriched by four rounds of *in vitro* selection. In the first two rounds MDA-MB-231-ErbB2 cells, a kind gift from Dr. Maria Jaramillo, NRC Canada, were used as the antigen. Three wells each with 1.5 million cells were incubated with 5×10^12^ phages displaying V_H_. For the 3^rd^ and 4^th^ round of panning human HER2 (ErbB2)/Fc chimera (R&D Systems Inc, Minneapolis, MN) at the concentration of 100 µg mL^−1^ was used. 100 mM triethylamine at pH 11.0 was used to elute bound phage which were immediately neutralized with 1 M Tris-HCl at pH 7.4 and were used to infect exponentially growing TG1 cells.

Individual colonies obtained after fourth round of panning were tested against HER2 in a phage ELISA according to the standard procedures. Briefly, clones were grown in 2 × YT medium + ampicillin (100 mg mL^−1^) 0.1% glucose to OD_600_  =  0.3–0.5, and infected with M13K07 helper phage (37°C no shaking, 30 min) followed by addition of kanamycin (50 µg mL^−1^) and incubation overnight (37°C with shaking). Cultures were centrifuged (4000 rpm, 20 min, 4°C) to pellet the cells. Subsequently 100 µL of supernatant containing recombinant phage particles were added to HER2 (ErbB2)/Fc chimera pre-coated microtiter plate wells. After a 2 h incubation at 37°C, microtiter plate wells were washed three times with PBS plus 0.1% (v/v) Tween 20 followed by addition of 1∶5000 diluted anti-M13 HRP conjugate (GE Healthcare, Baie d'Urfé, QC). V_H_-phages bound to HER2 were detected by the addition of 100 µL of HRP substrate. After a 15 min incubation the reaction was stopped by adding 100 µL of 1 M H_3_PO_4_ and absorption at 405 nm was measured.

### Expression and purification of sdAbs

The V_H_ genes of the clones that scored positive in phage ELISA were sequenced to examine the diversity of the isolated clones. They were re-cloned into the expression vector pSJF2H ^30^ using the restriction enzymes BbsI and BamHI. The ligated plasmids were used to transform *E. coli* cells. After sequence re-confirmation, recombinant sdAbs proteins were expressed in the periplasm and purified by immobilized-metal affinity chromatography (IMAC). Briefly, clones were inoculated overnight in 25 mL LB with 100 µg mL^−1^ ampicillin at 37°C and 200 rpm. Twenty milliliter of the culture was transferred to 1 L of M9 medium (0.2% glucose, 0.6% Na_2_HPO_4_, 0.3% KH_2_PO_4_, 0.1% NH_4_Cl, 0.05% NaCl, 1 mM MgCl_2_, 0.1 mM CaCl_2_) supplemented with 0.4% casamino acids, 5 µg mL^−1^ vitamin B1 and 100 µg mL^−1^ ampicillin and incubated for 24 h. Hundred milliliter of 10 × TB nutrients (12% Tryptone, 24% yeast extract and 4% glycerol), 2 mL of 100 µg mL^−1^ ampicillin and 1 mL of 1 M isopropyl-β-D-thiogalactopyranoside (IPTG) were added to the culture and incubation was continued for another 65 - 70 h at 28°C and 200 rpm. Cells were then centrifuged and the pellets were lysed with lysozyme. Cell lysates were centrifuged and supernatants were loaded onto 5-mL HiTrap^TM^ chelating HP affinity columns (GE Healthcare). After washing the columns with four column volume of wash solution (10 mM HEPES buffer, 500 mM NaCl, 20 mM imidazol, pH 7.5), His-tagged proteins were eluted with a linear gradient (10 to 500 mM) of imidazole and the eluted proteins were assessed for purity by SDS-PAGE and subsequently dialyzed in PBS buffer. The aggregation status of sdAbs was assessed by Superdex™ 75 size exclusion chromatography (SEC) as described using PBS as the equilibration buffer [31].

### Thermostability determination of sdAbs

To determine the circular dichroism (CD) profile and thermostability of the sdAbs, proteins were first buffer-exchanged in 10 mM phosphate buffer, pH 7.0, by a Superdex^TM^ 75 SEC. The peaks for the sdAbs were collected, and the proteins were used for CD analysis. CD spectra were collected from 250 to 200 nm at protein concentrations of 2.5 µM in a 10 mm quartz cuvette with a J-850 CD spectropolarimeter (JASCO, Easton, MD). Data were collected with a band width of 1.0 nm and a scanning speed of 50 nm min^−1^ with two accumulations of scans to determine the CD profile of the proteins. Under the same conditions but with a single data accumulation, CD spectra were automatically measured at 2°C intervals from 25–91°C to determine thermal denaturation of the protein at a temperature shift speed of 1°C/min. Ellipicity at 217 nm were plotted against temperature and melting temperatures (T_m_s) were calculated from Boltzmann Sigmoidal equation using GraphPadPrism software.

### Affinity measurement of sdAb by surface plasmon resonance (SPR)

The binding of sdAbs to HER2 was determined by SPR using a Biacore 3000 (GE Healthcare). HER2 and ovalbumin (as reference protein, Sigma) were immobilized on research grade sensor chip CM5 (GE Healthcare). Immobilizations were performed with an amine-coupling kit (GE Healthcare) and carried out at 50 µg mL^−1^ HER2 in 10 mM acetate, pH 4.0 (GE Healthcare) and 50 µg mL^−1^ of ovalbumin in 10 mM acetate, pH 4.5. A volume of 120 µL of the sdAb at concentration of 5 nM to 600 nM were injected over the surfaces at a flow rate of 40 µL/min. Analyses were carried out at room temperature in HBS-EP (10 mM HEPES pH 7.4, 150 mM NaCl, 3 mM EDTA and 0.005% surfactant P20 (GE Healthcare). Regeneration was performed with running buffer (HBS-EP). Data were analyzed with BIAevaluation software 4.1 (GE Healthcare).

### Crystallization and data collection

Various commercial kits of Hampton Research and Emeralds BioSystems were used for initial screening of crystallization conditions for Gr6 protein, by vapor diffusion method performed with a Honeybee 961 robot (Digilab Genomic Solutions). Gr6 crystals were obtained in sitting drops mixing 0.5 µL of protein (30 mg mL^−1^ in 10 mM HEPES pH 7.0, 100 mM NaCl) with 0.5 µL of precipitant solution (100 mM Bicine buffer, pH 9.0, 20% (w/v) PEG 6000). Temperature was an important factor for the growth of Gr6 crystals. The plate-shaped crystals grew in the way that crystallization trays were incubated at 25°C for 2 days, and then shifted to 4°C. Crystals grew to maximum dimension ∼ 0.30×0.50×0.03 mm within 2 weeks. Crystals in the mother liquor were flash-frozen in liquid nitrogen prior to data collection.

The native data set was collected to 1.6 Å resolution with the wavelength λ = 1.0 Å at beamline 13B1 of the National Synchrotron Radiation Research Center, Hsinchu, Taiwan. Crystals were kept at 100 K during data collection. Data were indexed, integrated and scaled with HKL2000 [32]. The space group is P2_1_2_1_2_1_ with the unit cell dimensions a = 39.81 Å, b = 76.99 Å and c = 81.36 Å. The Matthew's coefficient is 2.34 Å^3^/Da with two molecules per asymmetric unit corresponding to solvent content of 47.4%. Crystal parameters and data collection statistics are summarized in Table 1.

### Structure determination and refinement

The Gr6 structure was solved by molecular replacement with the program Phaser [33], using data between 20 and 2.5 Å. The monomer structure of the camelized human antibody fragment cV_H_-E2 (PDB code 1OL0) [21] was used as the search model and two copies of the molecule were applied in the search. The best solution from Phaser had a log-likelihood gain of 805.3. The model was rebuilt with the program Coot [34] with the guidance of the 2F_o_-F_c_ and F_o_-F_c_ electron density maps. Refinement of the model was carried out with the program CNS [35] without any non-crystallographic symmetry restraints. Multiple cycles of simulated annealing, positional, and individual isotropic B factor refinements against data to 1.6 Å resolution were performed with CNS, alternating with manual model rebuilding in Coot. 6.7% of the data were flagged for cross-validation during refinement. Total of 266 water molecules were included in the final model and final stage of the refinement. The refinement statistics are summarized in Table 1.

### PDB accession code

Coordinates and structure factors have been deposited in the Protein Data Bank with the identifier 3QYC.

## References

[1] Padlan EA (1994). Anatomy of the antibody molecule.. Mol Immunol.

[2] Williamson AR (1976). The biological origin of antibody diversity.. Annu Rev Biochem.

[3] Schroeder HW, Cavacini L (2010). Structure and function of immunoglobulins.. J Allergy Clin Immunol.

[4] Hamers-Casterman C, Atarhouch T, Muyldermans S, Robinson G, Hamers C (1993). Naturally occurring antibodies devoid of light chains.. Nature.

[5] Wesolowski J, Alzogaray V, Reyelt J, Unger M, Juarez K (2009). Single domain antibodies: promising experimental and therapeutic tools in infection and immunity.. Med Microbiol Immunol.

[6] Arbabi Ghahroudi M, Desmyter A, Wyns L, Hamers R, Muyldermans S (1997). Selection and identification of single domain antibody fragments from camel heavy-chain antibodies.. FEBS Lett.

[7] Behar G, Siberil S, Groulet A, Chames P, Pugniere M (2008). Isolation and characterization of anti-FcgammaRIII (CD16) llama single-domain antibodies that activate natural killer cells.. Protein Eng Des Sel.

[8] Dumoulin M, Conrath K, Van Meirhaeghe A, Meersman F, Heremans K (2002). Single-domain antibody fragments with high conformational stability.. Protein Sci.

[9] Stijlemans B, Conrath K, Cortez-Retamozo V, Van Xong H, Wyns L (2004). Efficient targeting of conserved cryptic epitopes of infectious agents by single domain antibodies. African trypanosomes as paradigm.. J Biol Chem.

[10] Behar G, Chames P, Teulon I, Cornillon A, Alshoukr F (2009). Llama single-domain antibodies directed against nonconventional epitopes of tumor-associated carcinoembryonic antigen absent from nonspecific cross-reacting antigen.. FEBS J.

[11] Baral TN, Magez S, Stijlemans B, Conrath K, Vanhollebeke B (2006). Experimental therapy of African trypanosomiasis with a nanobody-conjugated human trypanolytic factor.. Nat Med.

[12] Cortez-Retamozo V, Lauwereys M, Hassanzadeh Gh G, Gobert M, Conrath K (2002). Efficient tumor targeting by single-domain antibody fragments of camels.. Int J Cancer.

[13] To R, Hirama T, Arbabi-Ghahroudi M, MacKenzie R, Wang P (2005). Isolation of monomeric human V(H)s by a phage selection.. J Biol Chem.

[14] Jespers L, Schon O, James LC, Veprintsev D, Winter G (2004). Crystal structure of HEL4, a soluble, refoldable human V(H) single domain with a germ-line scaffold.. J Mol Biol.

[15] Arbabi-Ghahroudi M, MacKenzie R, Tanha J (2009). Selection of non-aggregating VH binders from synthetic VH phage-display libraries.. Methods Mol Biol 525: 187-216,.

[16] Barthelemy PA, Raab H, Appleton BA, Bond CJ, Wu P (2008). Comprehensive analysis of the factors contributing to the stability and solubility of autonomous human VH domains.. J Biol Chem.

[17] Arbabi-Ghahroudi M, To R, Gaudette N, Hirama T, Ding W (2009). Aggregation-resistant VHs selected by in vitro evolution tend to have disulfide-bonded loops and acidic isoelectric points.. Protein Eng Des Sel.

[18] Els Conrath K, Lauwereys M, Wyns L, Muyldermans S (2001). Camel single-domain antibodies as modular building units in bispecific and bivalent antibody constructs.. J Biol Chem.

[19] Kabat EA, Wu TT (1991). Identical V region amino acid sequences and segments of sequences in antibodies of different specificities. Relative contributions of VH and VL genes, minigenes, and complementarity-determining regions to binding of antibody-combining sites.. J Immunol.

[20] Vaguine AA, Richelle J, Wodak SJ (1999). SFCHECK: a unified set of procedures for evaluating the quality of macromolecular structure-factor data and their agreement with the atomic model.. Acta Crystallographica Section D.

[21] Dottorini T, Vaughan CK, Walsh MA, LoSurdo P, Sollazzo M (2004). Crystal structure of a human VH: requirements for maintaining a monomeric fragment.. Biochemistry.

[22] Fan Z-c, Shan L, Guddat LW, He X-m, Gray WR (1992). Three-dimensional structure of an Fv from a human IgM immunoglobulin.. Journal of Molecular Biology.

[23] Collaborative (1994). The CCP4 suite: programs for protein crystallography.. Acta Crystallographica Section D.

[24] Tina K, Bhadra R, Srinivasan N (2007). PIC: Protein interactions calculator.. Nucleic Acids Research.

[25] Willuda J, Honegger A, Waibel R, Schubiger PA, Stahel R (1999). High thermal stability is essential for tumor targeting of antibody fragments: engineering of a humanized anti-epithelial glycoprotein-2 (epithelial cell adhesion molecule) single-chain Fv fragment.. Cancer Res.

[26] Chatellier J, Van Regenmortel MH, Vernet T, Altschuh D (1996). Functional mapping of conserved residues located at the VL and VH domain interface of a Fab.. J Mol Biol.

[27] Vargas-Madrazo E, Paz-Garcia E (2003). An improved model of association for VH-VL immunoglobulin domains: asymmetries between VH and VL in the packing of some interface residues.. J Mol Recognit.

[28] Van Bockstaele F, Holz JB, Revets H (2009). The development of nanobodies for therapeutic applications.. Curr Opin Investig Drugs.

[29] Muyldermans S (2001). Single domain camel antibodies: current status.. J Biotechnol.

[30] Nahta R, Esteva FJ (2007). Trastuzumab: triumphs and tribulations.. *Oncogene*.

[31] Bell A, Wang ZJ, Arbabi-Ghahroudi M, Chang TA, Durocher Y (2009). Differential tumor-targeting abilities of three single-domain antibody formats..

[32] Otwinowski Z, Minor W (1997). Processing of X-ray diffraction data collected in oscillation mode.. Methods Enzymol.

[33] McCoy AJ, Grosse-Kunstleve RW, Adams PD, Winn MD, Storoni LC (2007). Phaser crystallographic software.. Journal of Applied Crystallography.

[34] Emsley P, Cowtan K (2004). Coot: model-building tools for molecular graphics.. Acta Crystallogr D Biol Crystallogr.

[35] Brunger A, Adams P, Clore G, DeLano W, Gros P (1998). Crystallography & NMR system: a new software suite for macromolecular structure determination.. Acta Crystallographica Section D: Biological Crystallography.

